# Effects of electroacupuncture frequencies on chronic low back pain in older adults: triple-blind, 12-months protocol for a randomized controlled trial

**DOI:** 10.1186/s13063-019-3813-6

**Published:** 2019-12-23

**Authors:** Sarina Francescato Torres, Ana Carolina Brandt de Macedo, Mateus Dias Antunes, Ingred Merllin Batista de Souza, Francisco Dimitre Rodrigo Pereira Santos, Adriana de Sousa do Espírito Santo, Flávia Ribeiro Jacob, Ariela Torres Cruz, Priscila de Oliveira Januário, Amélia Pasqual Marques

**Affiliations:** 1 School of Medicine, Department of Physical Therapy, Speech Therapy and Occupational Therapy, Postgraduate Program in Rehabilitation Sciences, University of Sao Paulo, Sao Paulo, Brazil; 2 Postgraduate Program in Rehabilitation Sciences, Department of Physical Therapy, Speech Therapy and Occupational Therapy, University of Sao Paulo, Rua Cipotânea, 51, São Paulo, São Paulo 05360-160 Brazil; 3 Department of Physical Therapy Prevention and Rehabilitation, Federal University of Parana, Parana, Brazil; 4 Higher Education Unit of the South of Maranhao, Maranhao, Brazil

**Keywords:** Acupuncture, Aged, Clinical trial, Electroacupuncture, Low back pain

## Abstract

**Background:**

Low back pain (LBP) is the most frequent complaint in clinical practice. Electroacupuncture treatment may be effective; however, the supporting evidence is still limited, especially in older adults.

**Objective:**

The current study is a randomized controlled trial that aims to evaluate the clinical efficacy of electroacupuncture in older adults with LBP.

**Methods:**

A five-arm randomized controlled trial with patients and evaluators blinded to the group allocation. A total of 125 participants with non-specific LBP will be randomly assigned into one of five groups: three electroacupuncture groups (low, high, and alternating frequency); one control group; and one placebo group. The electroacupuncture will be applied twice a week (30 min per session) for five weeks. The primary clinical outcome measure will be pain intensity. The secondary outcomes include: quality of pain; physical functioning; perceived overall effect; emotional functionality; patient satisfaction; and psychosocial factors. Patients will be evaluated before the first session, immediately after the last, and followed up after six and 12 months to check the medium- and long-term effects.

**Discussion:**

Although electroacupuncture is increasingly used to treat LBP, there is no guidance regarding the parameters used, which leads to inconsistent results. Thus, the effect of electroacupuncture (EA) on LBP remains controversial and requires more studies, especially in the older adult population.

**Conclusion:**

This is the first randomized controlled trial to evaluate the efficacy of different frequencies of electroacupuncture for treating chronic LBP in older adults. This study will provide evidence on the effectiveness of electroacupuncture as an alternative treatment method for LBP and will entail wider debate about an appropriate acupuncture intervention in this population.

**Trial registration:**

Clinicaltrials.gov, NCT03802045. Registered on 14 January 2019.

## Background

Low back pain (LBP) is the most frequent complaint in the clinical practice, with approximately 80% of the world population presenting with at least one episode throughout their lives [1]. Approximately six million older adults suffer from chronic LBP [2], with a prediction of increase in the coming years due to rapid and progressive population growth and aging [3]. These data are worrisome, since the elder population is the second most common age group to visit physicians due to LBP. This musculoskeletal condition is considered one of the main causes of disability worldwide, being strongly associated with functional incapacity, sleep disorders, withdrawal from social and recreational activities, psychological distress, cognitive deterioration, and falls in older adults [4, 5].

Pain is the main factor responsible for these harmful outcomes, therefore the treatment focuses mainly on analgesia and requires a combination of pharmacological and non-pharmacological therapies, where the latter should be emphasized [6]. Acupuncture is among the non-pharmacological strategies, the effects of which can be potentiated by electroacupuncture (EA). EA allows the stimulation of a larger area around the acupoint for a shorter time and, especially, allows parameters such as intensity, duration, and frequency of the stimulus to be easily identified and quantified [7].

However, the studies recommending EA as an effective and viable option for treating chronic LBP have methodological discrepancy regarding acupuncture techniques and procedures, with no guidelines regarding the standardization and efficiency of this treatment, which makes the healing effect of acupuncture controversial [8–10]. Hence, there is a need for rigorously studies that explore the acupuncture methods related with its therapeutic efficacy in order to improve validity of randomized clinical trials about acupuncture and EA in LBP [11].

Considering the prevalence of LBP in older people, the significance of health problems associated with this painful condition, and the lack of evidence in acupuncture guidelines, a thorough designed randomized controlled clinical trial is proposed.

### Study objectives

This study aims to evaluate the clinical efficacy of EA in older adults with LBP.

Our primary study goal is to screen a dominant EA frequency on pain management and its secondary outcomes in EA groups.

Our second goal is to determine whether EA is a more effective treatment than acupuncture (control) and placebo in older people with chronic LBP, in a one-year randomized controlled trial (RCT) (NCT03802045).

Our third goal is to determine the long-term effect of the EA, acupuncture, and placebo on LBP in older adults after six months and one year at the end of treatment.

### Trial design

This study is a five-arm clinical RCT with blinded patients and evaluators.

## Materials and methods

### Study setting

The study will be conducted at the University of São Paulo (USP) in partnership with the Federal University of Paraná (UFPR), both in Brazil. The study was approved by the Research Ethics Committee of the Medical School of USP (authorization no. 2.903.991) and financed with the authors’ own resources.

### Recruitment

Participants will be recruited through radio ads, social networks, and local newspapers. Individuals will be informed about the research proposal and treatment protocol, and those interested in participating will be selected according to the inclusion and exclusion criteria.

### Participant timeline

**Fig. 1:**
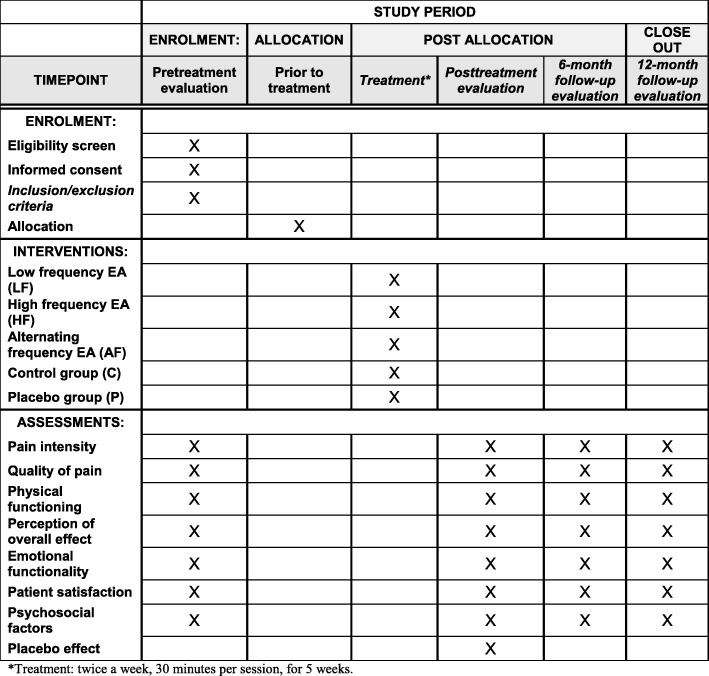
Study timepoints

The participant timeline is shown in Fig. 1.

### Inclusion criteria

We will use the World Health Organization (WHO) cut-off point of ≥ 60 years to refer to the elderly population in resource-poor countries [9]. Patients of both genders will be included. Other criteria are: (1) medical diagnosis of non-specific LBP of > 3 months’ duration; (2) with or without radiating leg pain; (3) a minimum pain intensity score of 4 on the 11-point pain numerical rating scale (NRS; Brazilian Portuguese version); (4) walk independently (with or without walking devices); and (5) signing of the consent form.

### Exclusion criteria

Patients will be excluded if: (1) they have had previous surgery on the spinal column; (2) have a known or suspected serious spinal pathology (e.g. cancer, vertebral fracture, spinal infection, or cauda equina syndrome); (3) fear of needles; (4) have participated in acupuncture treatment in the previous 30 days; or (5) if they are wheelchair users.

### Study standard

The study protocol is based on standards established under the Standards for Reporting Interventions in Clinical Trials of Acupuncture (STRICTA) and the Initiative on Methods, Measurement, and Pain Assessment in Clinical Trials (IMMPACT) [12].

### Randomization procedures

**Fig. 2:**
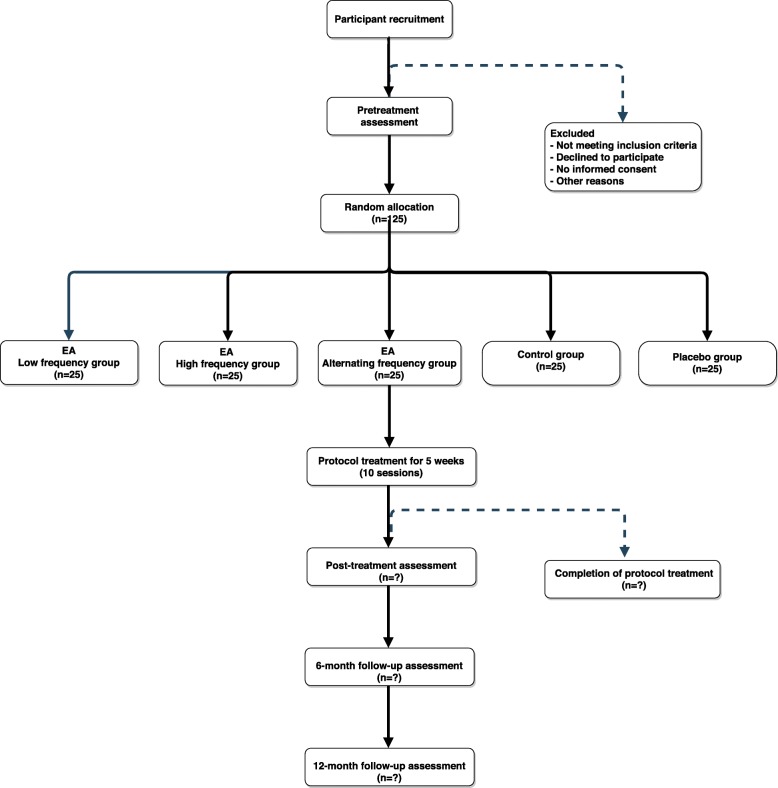
*Flow chart* of study design

The treatment allocation of each eligible individual in the study will be determined by a randomization process. The random allocation to the five arms of the study will be achieved using an online random-number generator (randomization.com) following a balanced 1:1 pattern with a block size of 5. This randomization process will be conducted by an independent staff person who is not involved in the study to guarantee concealment of the allocations. The information on the allocation list will remain strictly confidential. The allocations will be concealed and sequentially numbered; opaque, sealed envelopes will be used to contain the randomization assignments. The acupuncturist will open the envelopes according to the numerical sequence, immediately before the first session of treatment (Fig. 2).

### Blinding

Patients will remain blinded regarding the category of their allocation throughout the study data collection period. Additionally, the evaluators responsible for the data collection and the outcomes assessor will also be blinded to the patient allocation. The acupuncturist responsible for the interventions will be the only person not blind to the type of treatment to be performed.

### Qualification of practitioners

The interventions will be performed by acupuncture specialists with at least three years of clinical experience, who will receive prior training to ensure that they rigorously follow the study protocol and are familiar with the types of treatments, including details such as acupuncture points and manipulation of electroacupuncture parameters.

### Outcome measures

Four stages of evaluations will be performed: (1) before the start of treatment; (2) immediately after the end of treatment (five weeks); (3) at six months; and (4) one year after the final treatment. The evaluators will be trained in advance.

The primary clinical outcome will be pain intensity. The secondary outcomes will include quality of pain, physical functioning, perception of overall effect, emotional functionality, patient satisfaction, and psychosocial factors. All the scales and questionnaires have been translated into Brazilian Portuguese, with the exception of the visual analog scale for the assessment of anxiety, and their properties clinimetrically tested [13–16].

### Primary outcomes

#### Pain intensity

##### Numerical rating scale (NRS)

The NRS is an 11-point scale ranging from 0 (no pain) to 10 (worst possible pain). Participants will be asked to rate their average pain levels in the 24 h before the assessment [17].

The pressure pain threshold (PPT) will be measured using a pressure algometer (EMG System, Brazil). The assessed region will be marked with a tape measure and a dermatographic pencil. Two points will be marked bilaterally: 5 cm to the right and left of the spinous process of L3 and L5. A control point will be marked on the anterior tibial muscle of the right leg, 5 cm lateral to the tuberosity of the tibia [18]. The circular tip of the algometer will be positioned perpendicular to the participant’s skin and gradually pressed until the participant reports that the pressure has become painful and unbearable. Three measurements will be taken at each point with an interval of 1 min. Finally, the arithmetic mean will be used to define the pressure pain threshold [18].

### Secondary outcomes

#### Quality of pain

This will be analyzed using the McGill Pain Questionnaire, a valid and reliable instrument containing 78 pain descriptors divided into four categories (sensory, affective, evaluative, and miscellaneous) and 20 subcategories, to which scores of 1–5 are attributed [19, 20]. The result is obtained by first scoring the words according to their position within the set of descriptors of each subcategory. The maximum score in the sensory category is 42, in the affective 14, in the evaluative 5, and in the miscellaneous 17. The analysis is then performed by adding these values associated with their categories. Higher scores equate to more intense pain [19, 20].

#### Physical functioning

This measure will be assessed through the Roland Morris Disability Questionnaire (RMQ) and the Five Times Sit to Stand Test (FTSST).

RMQ is the Roland Morris Disability Questionnaire. It is a self-administered questionnaire, adapted to Brazilian Portuguese, which aims to evaluate physical incapacity due to LBP [13]. This contains 24 items pertaining to activities that can be impaired due to LBP. Individuals need to select the items that apply to their pain on that day. The selected items are summed for a total score ranging from 0 to 24, with higher scores indicating more severe functional incapacity

The activity of sitting and standing is a common and important movement in daily life; in clinical practice it helps to determine a person’s functional level [21]. The patient initiates the FTSST sitting in a chair without arm support, with the upper limbs crossed over the chest, feet positioned at hip width and knees in 90° flexion. They will be asked to stand and sit five times as fast as possible. A timer will be used to measure the task execution time. The test will be performed twice and the mean time will be calculated.

#### Perceived overall effect

The patient’s treatment self-perception will be evaluated using the Global Effect Perception Scale, an 11-point numerical scale ranging from − 5 (much worse) to + 5 (much better), where higher scores represent better recovery [17].

#### Emotional functionality

Depression and anxiety will be assessed using the Beck Depression Inventory (BDI) [14, 22] and the Global Anxiety - Visual Analog Scale (GA-VAS) [23], respectively.

The BDI is a self-administered questionnaire that contains 21 items, with a score in the range of 0–3, where a higher score indicates more depressive symptoms. The GA-VAS is composed of a 100-mm line, where the left extremity is related to the absence of anxiety and the right extremity is related to the worst possible anxiety. The individual is asked to assess the intensity of their anxiety in the previous 24 h and to mark this on the line. The distance from the left edge of the line to the mark placed by the patient is measured in millimeters. Greater measurements indicate greater anxiety.

#### Patient satisfaction

The evaluation of the patient’s treatment satisfaction will be carried out using the MedRisk questionnaire of patient satisfaction with physiotherapeutic care [15].

This questionnaire contains 20 items, covering global aspects of the treatment (two items), aspects related to the service provided (eight items), and aspects about the therapist–patient relationship (10 items). Following a Likert-type scale, the patient’s response will range from 1 (strongly disagree) to 5 (strongly agree), where higher scores represent greater satisfaction with the treatment.

#### Psychosocial factors

The risk of poor prognosis due to psychosocial factors will be assessed using the STarT Back Screening Tool (SBST), a self-administered questionnaire with nine items (four related to referred pain and five related to psychosocial factors), where the patient has the following response options: “I agree” (1 point) and “I Disagree” (zero points) for the first eight items; and “Nothing,” “A little,” or “Moderate” (zero points each), “A lot” or “Extremely” (1 point each) for the ninth item [16]. If the total score is in the range of 0–3 points, the patient is classified as low risk for poor prognosis. If the total score is > 3 points, the score of the psychosocial subscale, questions 5–9, is then considered. If the score of this subscale is ≤ 3 points, the patient is classified as medium risk and scores > 3 points are considered to indicate a high risk for poor prognosis [16].

### Procedures

After randomization, the acupuncturist will sequentially open the envelopes and the individuals will be randomly assigned to one of five treatment groups, each containing 25 participants: low frequency EA (LF); high frequency EA (HF); alternating frequency EA (AF); control group (C); and placebo group (P). The acupuncture points will be located and described according to the WHO Standard Acupuncture Locations [24]. Based on the beneficial effects of previous clinical trials, the acupoints selected for this study will be: BL23, BL25, BL40, SP6, and KI3 [10, 25–27] (Table 1).

**Table 1:** Protocol for acupoints

Acupoints	Location
BL23 (Shenshu)	In the lumbar region, at the same level as the inferior border of the spinous process of the second lumbar vertebra (L2). 1.5 B-cun lateral to the posterior median line.
BL25 (Dachangshu)	In the lumbar region, at the same level as the inferior border of the spinous process of the fourth lumbar vertebra (L4). 1.5 B-cun lateral to the posterior median line.
BL40 (Weizhong)	On the posterior aspect of the knee, at the midpoint of the popliteal crease.
SP6 (Sanyinjiao)	On the tibial aspect of the leg, posterior to the medial border of the tibia, 3 B-cun superior to the prominence of the medial malleolus.
KI3 (Taixi)	On the posteromedial aspect of the ankle, in the depression between the prominence of the medial malleolus and the calcaneal tendon.

*BL* bladder, *SP* spleen, *KI* kidney

### Interventions

Participants of the LF, HF, and AF groups will be submitted to the treatment protocol, which will consist of the bilateral application of electroacupuncture using a previously calibrated electrostimulator (Sikuro DS100C), consisted of alternating symmetrical biphasic waves with continuous pulse train for the low (2 Hz) or high (100 Hz) frequency groups, and a mixed pulse train for the alternating frequency group (100 Hz and 2 Hz for 3 s each); 100 ms pulse duration and 0.5 ms pulse width; and the maximum current (amplitude) intensity tolerated by the patient and intensified so that sensory habituation is avoided. Trichotomy will be carried out when necessary and the skin will be disinfected with 70% alcohol. With the participant lying down in a ventral position, the needles will be inserted at a 90° inclination with the skin, to a depth at which that the patient reports the “deQi” sensation (≅ 1.5 cm). The protocol sessions will happen twice a week (30 min per session) for five weeks, at no cost to the participants.

Sterile and disposable 0.25 × 30 mm stainless-steel needles (Dong Bang Acupuncture Inc., Seoul, Republic of Korea) will be used. The sessions will last 30 min, twice a week, for five weeks, totaling 10 sessions [10, 26].

The individuals that are randomly assigned to the C and P groups will follow exactly the same protocol as the electroacupuncture groups; however, the C group will not undergo electrical stimulation, as the acupuncturist will activate channels that are not connected to the patient. In the P group, an adhesive moxa (Dong Yang®) will be placed on each acupoint and the needle will be inserted over it, so that the participant only feels the needle prick, but without perforation of the skin and the “deQi” sensation. In addition, as in group C, the electrodes will be connected to the needles; however, no electrical current will be applied [28].

### Placebo effect

The individuals will be instructed since recruitment that this research has five groups with different types of treatment. However, to ensure the blinding of the participants and to avoid biases in measuring outcomes [11], they only will be enlightened about the control and placebo group when they finish the 12-month follow-up evaluation. Also, in the post-treatment evaluation, each one of them will be asked to answer the questions: “Did you feel the needle penetrate your skin?” and “Did you feel that you received real electroacupuncture?”

The percentage of yes and no answers will be analyzed. A significant percentage of “no” responses may suggest that the placebo effect was insufficient. According to Research Ethics Committee of the Medical School of University of São Paulo and Resolution 466/2012 of the Brazilian National Health Council [29], if we observe that the therapeutic effect of one group (EA treatment or control) will statistically greater than the placebo group, we will ensure that all participants of this group receive the best treatment when they finish the 12-month follow-up evaluation.

### Adverse events

At each treatment session, the participants will be asked about any unpleasant and unintended signs or symptoms associated with the use of electroacupuncture. A questionnaire will also be used with a record of the duration and intensity of the adverse symptom reported by the patient, which will be scored according to a Likert-type scale of 1–5, with 1 representing the absence of adverse symptoms and 5 severe adverse symptoms. The severe adverse symptoms will exclude the participant from the treatment and the participant will be referred for medical consultation.

### Withdrawal

Participants may withdraw from the study for any reason at any time, without penalty.

### Dropouts

All dropouts and attrition during the course of the study will be monitored and the respective reasons for withdrawal will be recorded. All randomized participants will be included in the analysis independent of whether they complete the intervention or not (intention-to-treat principles).

### Sample size

Based on a previous study [11], the sample size was calculated to detect a difference of 2 points on the pain intensity measured by the 11-point pain NRS. In our study, the sample size was calculated using the program G*Power 3 3.1. 9.2, with an estimated standard deviation of 1.47 points [30], a moderate effect size (0.35) [31], a statistical power of 80% (1 β error probability), and an α error level probability of 0.05. The study will require 105 individuals. However, an extra 20% of participants will be added in order to increase the power. Thus, this study will therefore require a total of 125 participants, with 25 in each group.

### Statistical analysis

The analysis of the data will follow the intention-to-treat principles. The normality of the data distribution will be assessed by the Kolmogorov–Smirnov test. The Pearson test will be used for correlations between variables that present a normal distribution, and the Spearman test will be adopted for correlations between variables that present a non-normal distribution.

The data will also be analyzed using the ANCOVA mixed model, considering age, gender, body mass index, and RMQ score as the covariates. The differences between groups for the primary and secondary outcome measurements will be analyzed using the linear mixed models (random intercepts and fixed coefficients), which incorporated terms for treatment, time, and the treatment by time interactions. The Bonferroni post hoc test will be used to control the overall type I error rate at the prespecified 5% level. Furthermore, in order to verify the magnitude of the differences between the interventions, the effect size (Cohen’s d) will be calculated [30].

All the analyses will be carried out using the SPSS program (IBM Corp., Armonk, NY, USA) for Windows, V.19.0. The confidence interval will be established at 95%, and the significance level will be set at 5%.

### Ethics and data security

All the patients will participate voluntarily and will sign a consent form before randomization. The access and storage of the data will be in accordance with the guidelines of the National Research Ethics Commission (CONEP). This study has been approved by the Ethics Committee of the University of São Paulo Medical School (authorization No. 2.903.991) and in ClinicalTrials.gov under number NCT03802045.

### Auditing

Data may be subject to audits, independent ethics committee (IEC)/Institutional Review Board (IRB) review and regulatory inspection(s). Local investigators will provide direct access to the source data documents.

## Results

This trial still has no results, since it is in the recruitment phase.

## SPIRIT

This protocol has been written in accordance with the Standard Protocol Items: Recommendations for Interventional Trials (SPIRIT) guidelines. The SPIRIT checklist is in Additional file 1.

## Discussion

Although acupuncture, both manual and electrical, is increasingly used to treat LBP, recent studies have concluded that its efficacy still needs to be established [32–35]. Discrepancies in its application and the low methodological quality of the studies produce inconsistent results and lead to it not being recommended according to the current clinical practice guidelines for patients with non-specific LBP [10, 32, 35]. Thus, with no guidance regarding the parameters used, the effect of EA on LBP remains controversial and requires more studies, especially in the older adult population, who are the leading population for this painful condition.

Therefore, there is a need for randomized, controlled, and well-delineated clinical trials in order to standardize EA treatment in LPB and to improve the level of scientific evidence. Here we describe the protocol for a clinical RCT to investigate the efficacy of different frequencies of EA for the treatment of older adults with chronic LBP, and thus to determine the most effective EA frequency.

We expect that the results of this study will aid in the standardization of the technique and provide convincing experimental evidence of the efficacy of EA treatment in older people with LBP.

Our study presents the non-blindness of the practitioner as a limitation, since it is not possible to blind the acupuncturist to the type of treatment.

### Trial status

This trial number NCT03802045 is currently in the recruitment phase. The date recruitment started was April 2019 and the expected date for recruitment completion is October 2019. (protocol version 2, 29 March 2019).

## Additional file

**Additional file 1.** SPIRIT-Checklist-for randomised studies.

## Data Availability

All data generated or analyzed in this study will be fully available without restriction through online platform on Clinicaltrials.gov (NCT03802045).

## Abbreviations

AF: Alternating frequency group
BDI: Beck Depression Inventory
C: Control group
CEP: Ethics Committee of the School of Medicine of the University of Sao Paulo
CONEP: National Commission for Research Ethics
EA: Electroacupuncture
FTSST: Five Times Sit to Stand Test
GA-VAS: Visual analog scale for the assessment of anxiety
GOV: Government
HF: High frequency group
ICC: Intraclass correlation
IMMPACT: Initiative on Methods, Measurement, and Pain Assessment in Clinical Trials
LBP: Low back pain
LF: Low frequency group
NRS: Numerical rating scale
P: Placebo group
PPT: Pressure pain threshold
RMQ: Roland Morris Disability Questionnaire
SBST: STarT Back Screening Tool
SPSS: Statistical Package for the Social Sciences
STRICTA: Revised Standards for Reporting Interventions in Clinical Trials of Acupuncture
UFPR: Federal University of Parana
USP: University of Sao Paulo
WHO: World Health Organization
